# Genetic and epigenetic divergence between disturbed and undisturbed subpopulations of a Mediterranean shrub: a 20‐year field experiment

**DOI:** 10.1002/ece3.2161

**Published:** 2016-05-09

**Authors:** Carlos M. Herrera, Pilar Bazaga

**Affiliations:** ^1^ Estación Biológica de Doñana Consejo Superior de Investigaciones Científicas (CSIC) Avenida Américo Vespucio s/n Isla de La Cartuja Sevilla 41092 Spain

**Keywords:** DNA methylation, ecological disturbance, ecological epigenetics, epigenetic diversity, genetic diversity, isolation by distance, spatial structure

## Abstract

Little is known on the potential of ecological disturbance to cause genetic and epigenetic changes in plant populations. We take advantage of a long‐term field experiment initiated in 1986 to study the demography of the shrub *Lavandula latifolia*, and compare genetic and epigenetic characteristics of plants in two adjacent subplots, one experimentally disturbed and one left undisturbed, 20 years after disturbance. Experimental setup was comparable to an unreplicated ‘Before‐After‐Control‐Impact’ (BACI) design where a single pair of perturbed and control areas were compared. When sampled in 2005, plants in the two subplots had roughly similar ages, but they had established in contrasting environments: dense conspecific population (‘Undisturbed’ subpopulation) versus open area with all conspecifics removed (‘Disturbed’ subpopulation). Plants were characterized genetically and epigenetically using amplified fragment length polymorphism (AFLP) and two classes of methylation‐sensitive AFLP (MSAP) markers. Subpopulations were similar in genetic diversity but differed in epigenetic diversity and multilocus genetic and epigenetic characteristics. Epigenetic divergence between subpopulations was statistically unrelated to genetic divergence. Bayesian clustering revealed an abrupt linear boundary between subpopulations closely coincident with the arbitrary demarcation line between subplots drawn 20 years back, which supports that genetic and epigenetic divergence between subpopulations was caused by artificial disturbance. There was significant fine‐scale spatial structuring of MSAP markers in both subpopulations, which in the Undisturbed one was indistinguishable from that of AFLP markers. Genetic differences between subpopulations could be explained by divergent selection alone, while the concerted action of divergent selection and disturbance‐driven appearance of new methylation variants in the Disturbed subpopulation is proposed to explain epigenetic differences. This study provides the first empirical evidence to date suggesting that relatively mild disturbances could leave genetic and epigenetic signatures on the next adult generation of long‐lived plants.

## Introduction

It is well‐known that ecological disturbances, defined as the punctuated killing or damaging of individuals that creates opportunities for new individuals to become established (Sousa 1984), can influence the functionality of ecosystems and the composition, spatio‐temporal dynamics and diversity of natural communities (Hooper et al. 2005; Villnäs et al. 2013; Eschtruth and Battles 2014; Huston 2014). Such ecosystem‐ and community‐level effects represent the aggregate outcome of species‐level responses, hence the importance of assessing the magnitude and understanding the mechanisms of individual species' responses to disturbance of their populations (Supp and Ernest 2014). In plants, substantial evidence demonstrates that disturbances can induce changes at both the individual (e.g., size, fecundity) and population levels (e.g., density, demography) (Cook and Lyons 1983; Herrera 1997; Pascarella and Horvitz 1998; Juenger and Bergelson 2000; Eschtruth and Battles 2014), which might in turn bring about rapid disturbance‐driven shifts in genotype composition (Scheiner and Teeri 1987). For example, among species that rely on a pre‐existing seed bank for reestablishment after fire or extended drought, biased genetic composition of the seed bank or selection on seedlings might lead to postdisturbance genetic shifts (Cabin et al. 1998; Dolan et al. 2008; Roberts et al. 2014). The potential of disturbance to cause rapid genetic change in plant populations and the possible mechanisms involved, however, remain essentially unexplored to date (Banks et al. 2013).

Epigenetic variation (based on, e.g., DNA cytosine methylation variants) can complement genetic variation (based on DNA sequence variants) as a source of phenotypic and functional variation in plants (Gao et al. 2010; Roux et al. 2011; Scoville et al. 2011; Medrano et al. 2014), and could also be involved in postdisturbance plant population responses. Virtually nothing is known, however, on the possible links between disturbance and epigenetic features of wild plant populations. Indirect support for positing a relationship between ecological disturbance and epigenetic characteristics of populations includes: (1) theoretical models documenting the significance of nongenetic inheritance systems for population persistence in fluctuating environments (Furrow and Feldman 2013; Geoghegan and Spencer 2013); (2) empirical results showing that epigenetic diversity may broaden the ecological niche and enhance the colonizing ability, expanding potential and resistance to perturbations of plant populations (Gao et al. 2010; Richards et al. 2012; Latzel et al. 2013; Medrano et al. 2014); and (3) increasing evidence showing that epigenetic mechanisms are involved in phenotypic plasticity (Herrera and Bazaga 2013; Jablonka 2013; Zhang et al. 2013), which in turn plays a facilitating role in the colonization of fluctuating environments (Herrera et al. 2012; Lande 2015). Furthermore, the flexibility and short‐term responsiveness of epigenetic variation to alterations in the biotic and abiotic environment (Gao et al. 2010; Lira‐Medeiros et al. 2010; Herman et al. 2013; Schulz et al. 2014), such as those ordinarily accompanying ecological disturbance, lend additional support to the hypothesis of disturbance‐mediated epigenetic changes in natural plant populations. This hypothesis, however, does not seem to have been explicitly addressed to date. Information on the epigenetic effects of disturbance will contribute to expand a bit further our knowledge of the manifold ecological implications of epigenetic mechanisms in natural plant populations (Bossdorf et al. 2008; Kilvitis et al. 2014).

Epigenetic and genetic variation may or may not be independent, and their ecological and evolutionary implications are also expected to differ (Richards 2006; Jablonka and Raz 2009; Herrera and Bazaga 2010; Jablonka 2013). Establishing the degree to which epigenetic variation is autonomous from genetic variation is therefore central to assessing the relevance of the former as an additional inheritance system (Richards 2006; Bossdorf et al. 2008). In addition, simultaneous consideration of epigenetic and genetic variation can provide a more realistic perspective on the mechanisms underlying the ecological consequences of disturbance (Herrera et al. 2016). This article presents an analysis of genetic and epigenetic correlates of disturbance in a wild‐growing population of the relatively long‐lived Mediterranean shrub *Lavandula latifolia* (Lamiaceae). The main aims of the study are (1) to assess the extent to which the genetic and epigenetic characteristics (diversity, composition, fine‐scale spatial structuring) of adult plants naturally reestablished after a disturbance differed from those of nearby adult plants established naturally under undisturbed conditions; (2) to evaluate the degree of independence of the genetic and epigenetic correlates of disturbance; and (3) to compare the magnitude and spatial pattern of postdisturbance differences in genetic and epigenetic diversity, which may shed light on the role of disturbance as both a selective agent and a releaser of epigenetic variation.

We take advantage here of a long‐term field experiment initiated in 1986 to study the demography of *L. latifolia* (Herrera 1991; Herrera and Jovani 2010), and compare genetic and epigenetic features of adult plants in two adjacent subplots, one experimentally disturbed and one left undisturbed, 20 years after disturbance. This span of time roughly matches the average longevity of reproductive individuals of the species (see below), which adds to the interest of the study. Research on effects of disturbance on plant population genetics either has mostly focused on short‐lived species or, when dealing with long‐lived ones, has considered only the earliest life stages (seeds, seedlings) and spanned over less than one generation (Parker et al. 2001; Dolan et al. 2008; Honnay et al. 2008; Roberts et al. 2014; but see Uchiyama et al. 2006). Our study design consisted of a single pair of treatment‐control subplots similar to unreplicated ‘Before‐After‐Control‐Impact’ (BACI) designs, where a single pair of perturbed and control sites are compared (Stewart‐Oaten et al. 1992; Miao et al. 2009). Limitations of BACI designs in general, and unreplicated ones in particular, have been thoroughly discussed in the ecological literature (Stewart‐Oaten et al. 1992; Osenberg et al. 1994; Stewart‐Oaten and Bence 2001; Payne 2006). There is no panacea to overcome these conceptual and statistical issues, but guiding statistical analyses by a priori hypotheses and plausible models and arguments, as will be done here, helps to mitigate them (Stewart‐Oaten et al. 1992; Stewart‐Oaten and Bence 2001). Furthermore, even though unreplicated designs fail at providing the data required for quantifying uncertainty and greatly limit generalizations, results from a single treatment‐control pair greatly reduce uncertainty relative to a prior state of no information (Grace et al. 2009). This holds true for the present study, which provides insights on genetic and epigenetic correlates of ecological disturbance for the first time.

## Materials and Methods

### Study plant

**Figure 1:**
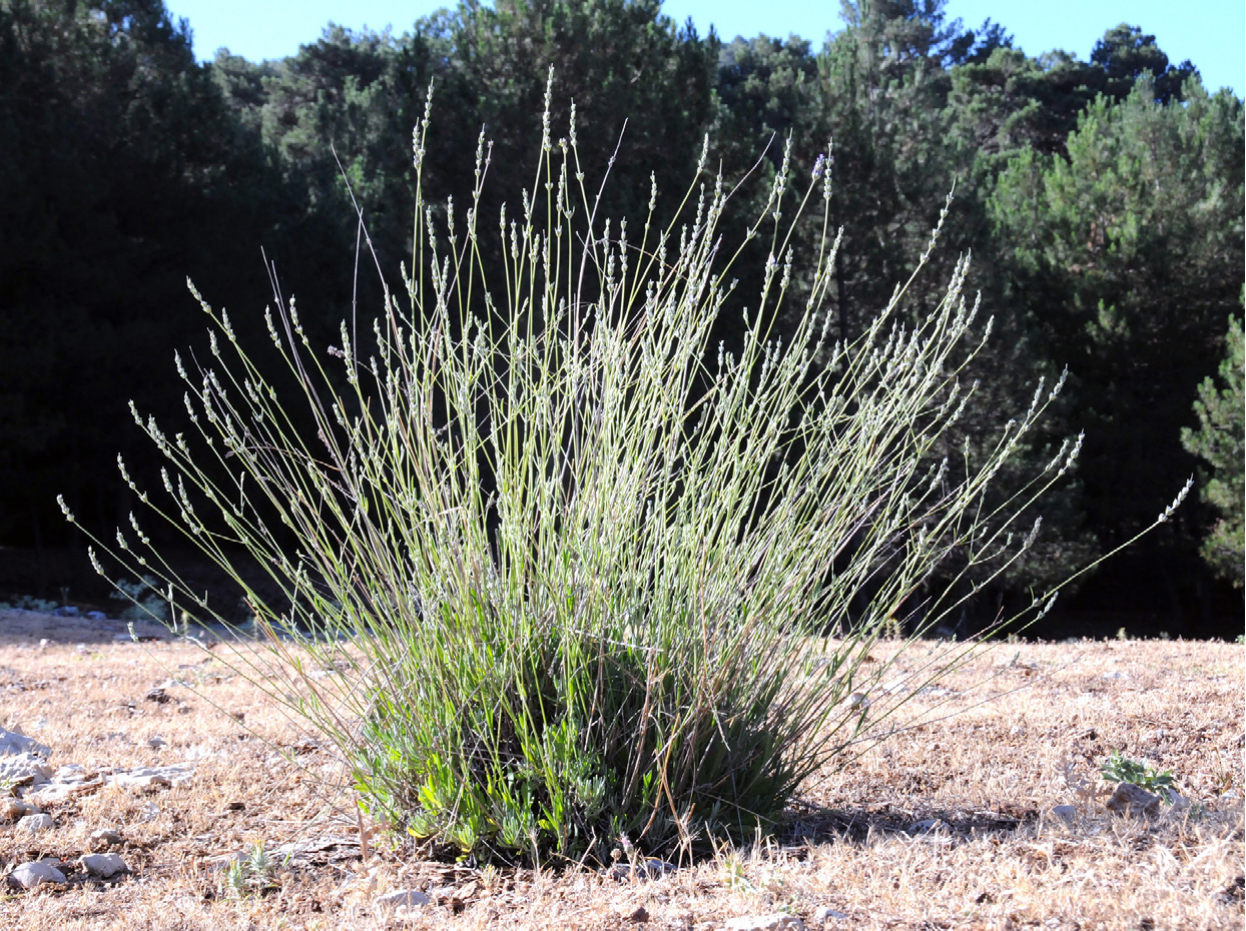
Flowering plant of *Lavandula latifolia*, a low evergreen shrub associated with clearings and well‐lit understory of conifer and mixed woodlands.

*Lavandula latifolia* is a low evergreen shrub (Fig. 1; see also appendix A in Herrera and Jovani 2010) characteristic of clearings and well‐lit undergrowth in open woodlands of the eastern Iberian Peninsula at 1000–1600 m a.s.l. In our Sierra de Cazorla study area (Jaén province, southeastern Spain), the ecological optimum for the species occurs around 1200–1300 m a.s.l. (Herrera and Bazaga 2008). Flowering lasts from July to October. Flowers are hermaphrodite, self‐compatible, and pollinated by a diverse assemblage of bees, butterflies, and flies (Herrera 1987a). The species reproduces exclusively by seeds, which are small (~ 1 mg) and lack special mechanisms for dispersal, falling passively to the ground after maturation. Most seeds disperse within 0.3 m of the edge of the parent's vertical projection (Herrera 1987b). The species lacks a persistent soil seed bank, as the vast majority of seeds germinate in the first few springs following dispersal. Seedling mortality during the first few summers is extensive, <6% remaining alive 6 year past emergence (Herrera 2000). In our study site (see below), plants flowered for the first time when 4–8 years old, mean longevity of individuals flowering at least once was 22 years, and only ~7% of these lived for >30 years (C. M. Herrera, unpubl. data).

### Study site and experimental population

This study was conducted at the ‘Aguaderillos‐2’ site of Herrera (1988, 1991), located at 1220 m elevation in a well‐preserved mixed woodland of *Pinus nigra* and *Quercus rotundifolia*. The understory was dominated by *L. latifolia*, which formed a continuous population over many hectares around the site. A permanent 20 × 20 m plot was established there in July 1986. The site was chosen because there was abundant ecological information on the local *L. latifolia* population (Herrera 1987a,b, 1991); vegetation had not experienced major alterations for at least 30 years, as judged from aerial photographs taken in 1956; recent minor natural disturbances (e.g., rock falls, wild boar uprootings) were not discernible; and the density and size distribution of *L. latifolia* plants within the plot were indistinguishable from those in the immediate surroundings. An arbitrary diagonal line was drawn that divided the square plot into two equal‐sized, right‐angle triangular subplots (~200 m^2^ each), and each half‐plot was randomly assigned to control and treatment experimental levels (‘Undisturbed’ and ‘Disturbed’ hereafter respectively).

All *L. latifolia* plants growing within the Disturbed subplot were removed by hand in July 1986. Particular care was taken to minimize disturbances of the upper soil layers. The subplot was thoroughly screened until all *L. latifolia* plants, including first year seedlings, had been removed. Surveys were later performed there on August 1989 and October 1990, and all new *L. latifolia* juveniles ≥1 year old found were mapped and individually tagged. Reestablishing plants were initially distributed fairly homogeneously over the Disturbed subplot, although spatially heterogeneous mortality eventually led to a clustered distribution. A demographic analysis for the period 1990–2008 of this naturally reestablished subpopulation was presented by Herrera and Jovani (2010). All *L. latifolia* plants within the Undisturbed subplot bearing ≥10 leaves (roughly ≥2 year in age) were mapped and tagged in August 1986. Preliminary demographic data for this subpopulation for the period 1986–1989 were presented in Herrera (1991; ‘Aguaderillos‐2 permanent plot’).

Analyses reported in this paper refer to the marked plants that remained alive in 2005 (66 and 84 plants in the Undisturbed and Disturbed subplots respectively). First‐year, undamaged mature leaves were collected from each plant in September 2005, placed in paper envelopes and dried immediately at ambient temperature in sealed containers with silica gel. Those sampled plants that died naturally during 2006–2014 were aged by ring counting (Herrera 1991). From these data, estimated mean age of sampled plants in 2005 in the Undisturbed and Disturbed subplots were 23 and 18 years respectively. The two groups of adult plants compared in this study therefore comprised individuals established within a relatively narrow temporal window (~1982–1987), their main difference involving the conspecific environments where establishment took place: mature, dense, highly‐competitive conspecific population (‘Undisturbed subpopulation’ hereafter) versus open space where competition for light, water, and nutrients had been suddenly released following the elimination of conspecifics (‘Disturbed subpopulation’ hereafter). Additional demographic information on the Disturbed and Undisturbed subpopulations during 1986–2005 relevant to this study will be summarized in Results.

### Laboratory methods

Genetic and epigenetic characteristics of the 150 *L. latifolia* plants sampled were assessed by fingerprinting them using amplified fragment length polymorphism (AFLP; Meudt and Clarke 2007) and methylation‐sensitive amplified polymorphism (MSAP; Schulz et al. 2013; Fulneček and Kovařik 2014) techniques. The MSAP technique is useful to identify genome‐wide methylation patterns in ecological epigenetics studies of species without detailed genomic information (Schrey et al. 2013). Frequent experimental demonstration that methylation status of MSAP markers often responds to environmental changes (Alonso et al. 2016) further motivates their use to investigate epigenetic consequences of disturbance.

Total genomic DNA was extracted from dry leaf samples using Qiagen DNeasy Plant Mini Kit (Qiagen, Valencia, CA, USA) and the manufacturer protocol. AFLP and MSAP analyses were conducted on the same DNA extracts. The AFLP analysis was performed using standard protocols involving the use of fluorescent dye‐labeled selective primers. Four *Eco*RI +> 3/*Mse*I + 3 and four *Pst*I +2/*Mse*I + 3 primer pair combinations were used that provided reliable, consistently scorable results, and each plant was fingerprinted using these combinations (Table S1). Fragment separation and detection was made using an ABI PRISM 3130xl DNA sequencer (Applied Biosystems, Foster City, CA, USA), and the presence or absence of each AFLP fragment in each individual plant was scored manually by visualizing electropherograms with GeneMapper 3.7 software (Applied Biosystems, Foster City, CA, USA). Only fragments ≥150 base pairs in size were considered to reduce possible biases arising from size homoplasy (Vekemans et al. 2002). AFLP genotyping error rates were determined for each primer combination by running repeated, independent analyses for 35 plants (23.5% of total), and estimated as the ratio of the number of discordant scores in the two analyses (all plants and markers combined) to the product of the number of plants by the number of scored markers. Average genotyping error rate (±SE) for the eight AFLP primer combinations used was 2.2 ± 0.3% (Table S1).

Methylation‐sensitive amplified polymorphism is a modification of the standard AFLP technique that uses the methylation‐sensitive restriction enzymes *Hpa*II and *Msp*I in parallel runs in combination with another restriction enzyme, commonly *Eco*RI or *Mse*I. *Mse*I was used here because of better repeatability of results (see also Herrera et al. 2013; Medrano et al. 2014). *Hpa*II and *Msp*I are isoschizomers that recognize the same tetranucleotide 5'‐CCGG but have differential sensitivity to methylation at the inner or outer cytosine. Differences in the products obtained with *Hpa*II and *Msp*I thus reflect different methylation states at the cytosines of the CCGG sites recognized by *Hpa*II or *Msp*I cleavage sites (Schulz et al. 2013; Fulneček and Kovařik 2014). MSAP assays for this study were conducted using four *Hpa*II‐*Msp*I + 2/*Mse*I + 3 primer combinations (Table S1). Fragment separation and detection was made using an ABI PRISM 3130xl DNA sequencer, and the presence or absence of *Hpa*II*/Mse*I and *Msp*I/*Mse*I fragments in each sample was scored manually by visualizing electropherograms with GeneMapper 3.7 software. MSAP genotyping error rates were estimated for each primer combination by running repeated *Hpa*II/*Mse*I and *Msp*I/*Mse*I analyses for 32 plants (21.5% of total), and computed as the ratio of the number of discordant scores in the two analyses (all plants and markers, and the two enzyme pairs, combined) to twice the product of the number of plants by the number of scored markers. Mean genotyping error rate (±SE) for the four MSAP primer combinations used was 3.1 ± 0.4% (Table S1).

### Data analysis

Two presence**–**absence matrices for MSAP fragments were obtained with the four *Hpa*II‐*Mse*I and *Msp*I‐*Mse*I primer combination pairs (Table S1). Different MSAP ‘scoring’ methods can be used to obtain from these data the sample x marker matrix containing information on the methylation status of cytosines at the CCGG end of fragments (MSAP ‘fingerprint’ matrix) (Herrera and Bazaga 2010; Schulz et al. 2013; Fulneček and Kovařik 2014). We used the ‘Mixed Scoring 2’ scheme of Schulz et al. (2013). The 173 MSAP fragments obtained (Table S1) were transformed into three distinct sets of markers corresponding to unmethylated (*u*‐type in Schulz et al. 2013 terminology), ^HMe^CG + ^Me^CG methylation (internal methylation plus hemimethylation, *m*‐type) and ^HMe^CCG methylation (external hemimethylation, *h*‐type) markers. Plants sampled were characterized epigenetically by means of the presence**–**absence scores for *u*‐type, *h*‐type and *m*‐type MSAP markers, using the Extract_MSAP_epigenotypes function from Schulz et al. (2013). The *u*‐type markers obtained (*N *=* *158) were nearly perfectly correlated with *m*‐type (*N *=* *122) and *h*‐type (*N *=* *153) ones (see also Medrano et al. 2014). For ease of interpretation only *h*‐ and *m*‐type markers will be considered in this study.

Unless otherwise stated, all statistical analyses were carried out using the R environment (R Development Core Team 2014). Three complementary, spatially nonexplicit methods were used to test for genetic and epigenetic differences between the Undisturbed and Disturbed subpopulations. First, analysis of molecular variance (AMOVA; Excoffier et al. 1992) was used to evaluate multilocus differentiation between subpopulations. Computations were conducted on the Jaccard pairwise dissimilarity matrix using the AMOVA function in the pegas package (Paradis 2010). The second method sought to identify individual AFLP and MSAP markers nonrandomly distributed between subpopulations. Marker score (presence**–**absence) × subpopulation (Undisturbed‐Disturbed) two‐way contingency tables were constructed for each marker, and *P*‐values obtained from Fisher exact‐probability tests were used to identify nonrandom marker‐subpopulation associations. Storey and Tibshirani's (2003) *q*‐value method was used to correct significance levels for multiplicity of statistical tests. The third method used random forests based on classification trees (Hastie et al. 2009) to identify markers relevant to predict individual subpopulation membership. Computations were performed with the Boruta package (Kursa and Rudnicki 2010). In contrast with marker‐by‐marker, independent marker‐subpopulation association tests, ‘all‐relevant feature selection’ performed by Boruta allowed the identification of markers with small, individually nonsignificant effects but significant interaction effects (Kursa and Rudnicki 2010). To minimize the effects of stochastic fluctuations (Calle and Urrea 2011), 300 repetitions of the Boruta analysis were run, and only markers confirmed as predictors of subpopulation membership in ≥95% of repetitions were deemed significant.

Whether genetic and epigenetic correlates of ecological disturbance were independent of each other was examined by testing whether (1) multilocus genetic and epigenetic variation was uncorrelated across plants; (2) multilocus epigenetic differences between subpopulations persisted after multilocus genetic differences were statistically accounted for; and (3) AFLP and MSAP markers nonrandomly distributed between subpopulations were uncorrelated. For testing (1), correlations were run between individual pairwise dissimilarity matrices for AFLP and MSAP markers, and statistical significance determined using Mantel tests. Test (2) was accomplished with partial Mantel tests, which involved estimating the correlation between two matrices while controlling for the effect of another (Legendre and Legendre 2012). Each dissimilarity matrix for MSAP markers was correlated with a binary‐coded pairwise distance matrix denoting if individuals in a pair belonged to the same or different subpopulations, while controlling for dissimilarity in AFLP markers. Computations for (1) and (2) were done with functions mantel and mantel.partial in package vegan (Oksanen et al. 2015), and significance levels were based on 10^5^ permutations. For test (3), AFLP marker score (presence‐absence) × MSAP marker score (presence‐absence) two‐way contingency tables were constructed for all possible pairs of markers whose frequencies differed significantly between subpopulations. The set of *P*‐values obtained from Fisher exact‐probability tests were used to identify possible AFLP and MSAP marker pairs exhibiting nonrandom covariation between subpopulations.

Differentiation between subpopulations could reflect some previous spatial gradient(s) in genetic and/or epigenetic composition across the plot, rather than population substructuring due to experimental disturbance (Meirmans 2012). This key aspect was scrutinized using two independent approaches. The first involved applying AMOVA to an artificial test data set where the diagonal to subdivide the plot into two subpopulations was drawn at a right angle to the original one. A causal effect of disturbance should lead to consistently lower differentiation between artificial test groups than between the Disturbed and Undisturbed subpopulations. The second approach examined whether AFLP and MSAP markers nonrandomly distributed between subpopulations defined two distinct spatial clusters of individuals separated by an abrupt transition matching the line of demarcation between subplots drawn in 1986. Following Blair et al. (2012), the spatially explicit Bayesian model implemented in version 4.0.4 of the Geneland package (Guillot et al. 2005; Guillot and Santos 2010) was used to cluster individuals into two groups (*K *=* *2). Then we assessed whether the resulting cluster memberships were actually separated by the linear boundary between subplots, as would be expected if observed genetic and epigenetic divergence between subpopulations was caused by disturbance. Fifty independent runs of the MCMC function were run, and maps of posterior probabilities of cluster membership for each pixel of the spatial domain were obtained for the model with the lowest mean log posterior probability.

Possible mechanisms underlying the genetic and epigenetic differences between plants of the two subpopulations were explored by examining differential fit of genetic and epigenetic data to isolation‐by‐distance models (Guillot et al. 2009; Herrera et al. 2016). Comparisons of isolation‐by‐distance patterns exhibited by plant pairs from the same and different subpopulations can also help to set apart differentiation between subpopulations attributable to pre‐existing isolation by distance and differentiation following experimental disturbance (Guillot et al. 2009; Ley and Hardy 2013, 2014). Isolation by distance was tested by examining whether the slope of the regression between pairwise genetic/epigenetic similarity between individuals and pairwise spatial distance (log‐transformed) was negative and significantly different from zero (Rousset 1997, 2000). This method does not require classification of pairwise distance data into arbitrary intervals, which makes it robust to the spatially clustered distribution of plants in the Disturbed subpopulation. The kinship coefficient between pairs of individuals was computed using the estimator proposed by Hardy (2003) for dominant markers, assuming an inbreeding coefficient of 0.1 (C. M. Herrera, unpubl. data) and using all individuals in the sample for defining reference allele frequencies. Differences between genetic markers in heritability, selection and mutation rates are expected to produce contrasting isolation‐by‐distance patterns (Epperson 1990, 2005; Ley and Hardy 2013; Herrera et al. 2016). Independent analyses were thus conducted for AFLP, MSAP *h*‐type and MSAP *m*‐type markers. SPAGeDi version 1.4 (Hardy and Vekemans 2002) was used for computing kinship estimates and kinship‐log distance regression slopes. The latter's statistical significance was tested by permutations with 5000 repetitions.

## Results

### Undisturbed and disturbed subpopulations, 1986–2005

**Figure 2:**
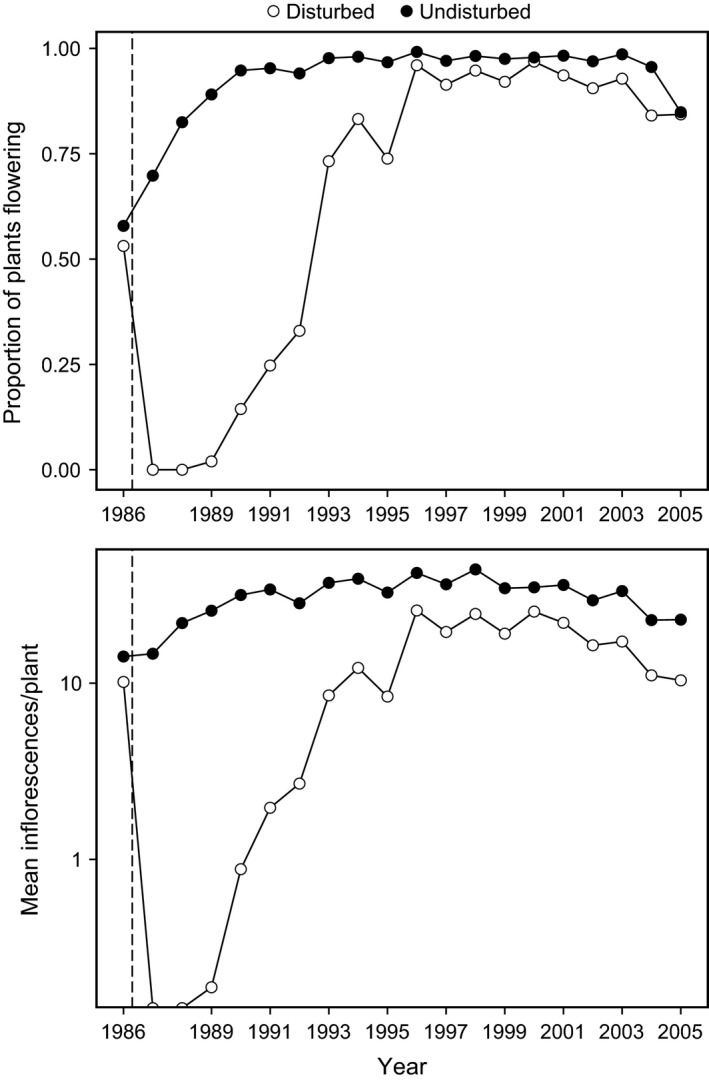
Dynamics over 1986–2005 of the proportion of *Lavandula latifolia* plants producing inflorescences (upper graph), and mean number of inflorescences produced per plant (a proxy for plant size, lower graph), in the Undisturbed and Disturbed subplots. The vertical dashed line marks the time of experimental removal of all *L. latifolia* plants from the Disturbed subplot, after which a new subpopulation reestablished there naturally. Plants were sampled for this study in 2005.

Prior to experimental disturbance, *L. latifolia* plants in the two contiguous subplots were similar in size and reproductive features, as shown by comparisons of the proportion of plants bearing inflorescences and number of inflorescences per plant (Fig. 2), which is closely correlated with plant size in this species (Herrera 1991; Herrera and Jovani 2010). In the Undisturbed subplot, frequency of inflorescence‐bearing individuals and mean number of inflorescences per plant increased steadily until 1996, and then remained fairly stable until 2005 (Fig. 2). In the Disturbed subplot, the most precocious newly established individuals flowered for the first time in 1990. Proportion of plants flowering and mean inflorescences per plant increased there steeply during the following years until reaching a plateau in 1996 (Fig. 2). By 1996, the reproductive parameters of marked plants in the Disturbed subplot had already converged with those in the Undisturbed subplot, and subsequently the two subpopulations remained similar and exhibited parallel annual fluctuations until 2005 (Fig. 2).

### Genetic and epigenetic variation

Plants sampled in 2005 were fingerprinted using 230 AFLP and 173 MSAP fragments (Table S1). The majority of MSAP fragments (85.0%) produced *h*‐ or *m*‐type markers, and a total of 275 *h*‐ and *m*‐type markers were obtained (153 and 122, respectively; Table S1). For all plants combined, 43.0% of AFLP markers and 53.5% of MSAP markers were polymorphic (at least 2% of samples showed a variant score; Table S1), the difference between marker types being statistically significant (*P *=* *0.02; Fisher exact‐probability test). Epigenetic diversity also exceeded genetic diversity when subpopulations were considered separately, the difference being particularly marked in the Disturbed one (Table 1).

**Table 1:** Genetic and epigenetic diversity of *Lavandula latifolia* plants in the Undisturbed and Disturbed subplots, as estimated by percent polymorphism of AFLP and MSAP markers. A marker was considered polymorphic if >2% of samples showed a variant score. Limits of 95% binomial confidence interval of polymorphism estimates shown in parentheses

Marker type	Subpopulation		*P*‐value^a^
	Undisturbed	Disturbed	
AFLP markers (*N *=* *230)	34.6 (28.6–41.1)	39.3 (33.1–45.9)	0.34
MSAP markers			
All (*N *=* *275)	45.1 (39.1–51.1)	70.2 (64.3–75.4)	<0.00001
*m*‐type (*N *=* *122)	53.3 (44.0–62.3)	65.6 (56.3–73.8)	0.067
*h*‐type (*N *=* *153)	38.6 (30.9–46.8)	73.8 (66.0–80.5)	<0.00001

a Statistical significance of difference tested with Fisher exact‐probability tests.

Epigenetic variation was (statistically) unrelated to genetic variation across plants, as shown by negligible and statistically nonsignificant correlations between the pairwise dissimilarity matrix for AFLP markers and each of the corresponding dissimilarity matrices for all (*r *= −0.045, *P *=* *0.82), *m*‐type (*r *=* *−0.023, *P *=* *0.68), and *h*‐type MSAP markers (*r *=* *0.022, *P *=* *0.19). The contrasting pattern of variation between subpopulations in genetic and epigenetic diversity (Table 1) thus most likely reflects the independence of genetic and epigenetic variation in the sample of plants studied. Subpopulations did not differ significantly in genetic diversity, but they did differ markedly in epigenetic diversity, with MSAP marker polymorphism being much higher in the Disturbed (70%) than in the Undisturbed subpopulation (45%) (Table 1). Although the two MSAP marker types exhibited similar diversity trends, the difference between subpopulations was greatest for *h*‐type markers (Table 1). Only polymorphic AFLP (*N *=* *99) and MSAP (*N *=* *147) markers will be considered in subsequent analyses.

### Genetic and epigenetic differences between subpopulations

Analysis of molecular variances revealed significant genetic (AFLP) and epigenetic (MSAP) differences between subpopulations (Table 2), which accounted for ~2.5% and ~1.5% of total genetic and epigenetic variance in the whole sample respectively. Separate analyses of the two MSAP marker types indicated that epigenetic differences between subpopulations were mainly due to differentiation in *m‐*type markers (Table 2). Differentiation in *h*‐type markers was weaker and did not reach statistical significance. Irrespective of marker type, differentiation between groups in the artificial test data set (created by dividing the plot by a diagonal perpendicular to the actual line of subpopulation demarcation) was consistently smaller than differentiation between Disturbed and Undisturbed subpopulations (Table 2).

**Table 2:** Genetic and epigenetic differentiation (Φ_ST_) between plants in the Undisturbed and Disturbed subplots (‘Actual data’), estimated by applying analysis of molecular variance (AMOVA) to individual pairwise dissimilarity matrices. ‘Artificial test data’ were obtained by bisecting the plot with a diagonal line perpendicular to the diagonal that truly separated the Disturbed and Undisturbed subplots

Marker type	Actual data		Artificial test data	
	Φ_ST_	*P*‐value^a^	Φ_ST_	*P*‐value^a^
AFLP	0.025	0.0002	0.001	0.50
MSAP				
All	0.014	0.0012	0.004	0.32
*m*‐type	0.019	0.0007	0.008	0.05
*h*‐type	0.005	0.32	0.000	0.76

a Permutation‐based tests with 10^5^ repetitions. *P*‐values shown were computed by applying the Holm‐Bonferroni correction for multiple tests to the original *P*‐values resulting from permutation tests.

Epigenetic divergence between subpopulations was unrelated to genetic differences. After controlling for genetic differences (partialling on the AFLP dissimilarity matrix), dissimilarity matrices based on all and *m*‐type MSAP markers were still significantly correlated with the binary matrix of subpopulation membership (*r *=* *0.037 and 0.035, respectively; *P *=* *0.009, partial Mantel tests). The dissimilarity matrix based on *h*‐type MSAP markers (which did not differ between subpopulations) was uncorrelated with subpopulation membership after controlling for genetic dissimilarity (*r *=* *−0.002, *P *=* *0.53).

**Figure 3:**
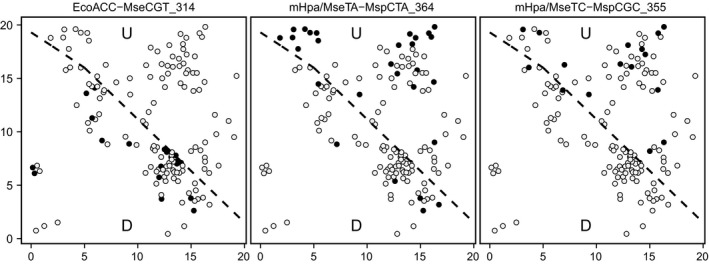
Maps of the 20 × 20 m study plot showing the spatial distribution of the *Lavandula latifolia* plants sampled (dots) and their scores for three representative AFLP and MSAP markers nonrandomly distributed between subpopulations (see Table S2 for complete list and marker naming conventions). Marker presence–absence for a given plant is coded as filled and empty dots respectively. The diagonal dashed lined denotes the boundary between the Undisturbed (U, upper right sector in each map) and Disturbed (D, lower left sector) subplots. Coordinates on axes are in meters.

Several AFLP and MSAP markers were nonrandomly distributed between subpopulations, as revealed by both marker x subpopulation association tests and Boruta analyses (Table S2). The marker × subpopulation association tests revealed that the frequency of six AFLP (out of 99 tested, 6.1%) and five *m*‐type MSAP (out of 71 tested, 7.0%) markers differed significantly between subpopulations. Among *h*‐type MSAP markers, none was found to be nonrandomly distributed between subpopulations. The Boruta analysis identified three AFLP and four MSAP markers with significant predictive value to discriminate between subpopulations (Table S2). With the single exception of an *h*‐type MSAP marker, all of these were also shown by marker × subpopulation association analyses to be nonrandomly distributed between subpopulations (Table S2). Unequal distribution between subpopulations of three representative discriminatory markers is illustrated in Figure 3. For five of the six MSAP markers nonrandomly distributed among subpopulations (Table S2), there was a consistent trend toward lower methylation frequency in the Disturbed than in the Undisturbed subpopulation (results not shown). None of the 36 association tests between the six AFLP and six MSAP markers nonrandomly distributed between subpopulations was statistically significant (*P *≥* *0.36; Fisher exact‐probability tests), which further supports the independence of genetic and epigenetic differences between subpopulations.

**Figure 4:**
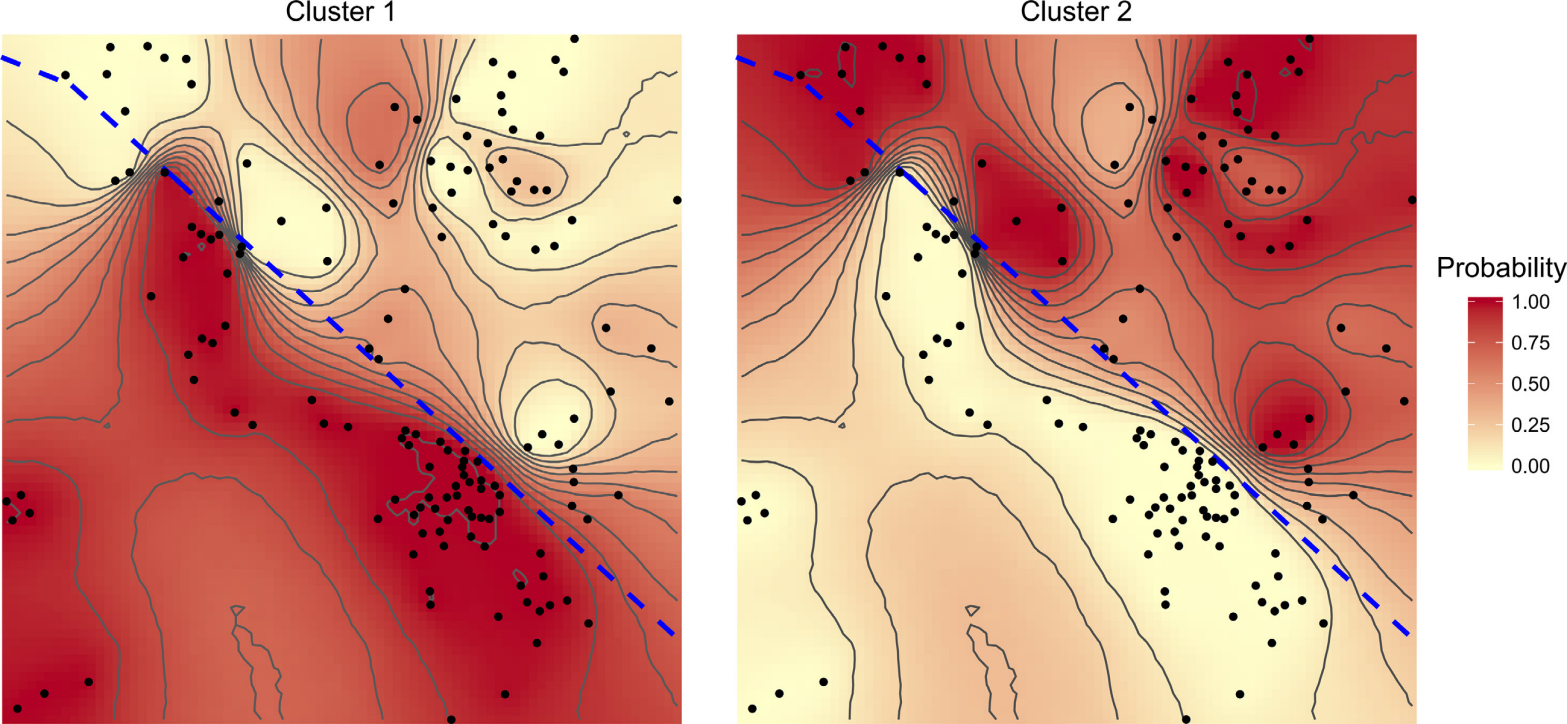
Contour maps showing the posterior probabilities of belonging to clusters 1 and 2 obtained by application of spatial Bayesian clustering, using *K *=* *2 and AFLP and MSAP data for markers nonrandomly distributed between subpopulations (Table S2). Dots mark the position of plants, and the thick diagonal dashed line indicates the demarcation between Undisturbed (upper right) and Disturbed (lower left) subplots drawn at the beginning of the study, 20 years before adult plants were sampled for genetic and epigenetic analyses.

Bayesian clustering on AFLP and MSAP markers nonrandomly distributed between subpopulations (Table S2) defined two distinct spatial clusters of individuals whose geometry and distribution matched Disturbed and Undisturbed subpopulations (Clusters 1 and 2 respectively; Fig. 4). Contour maps of posterior probabilities of cluster membership revealed an abrupt linear transition between clusters whose location was closely coincident with the demarcation line between subplots drawn at the beginning of the study (Fig. 4).

### Spatial structure of genetic and epigenetic variation

Genetic and epigenetic variation were spatially structured at the 20 × 20 m scale of our study plot, but the strength of isolation by distance varied across marker types and plant pair classes (Table 3). For AFLP markers, slopes of kinship‐log distance regressions were negative and statistically significant irrespective of whether plant pairs were in the same or different subpopulations; for *m*‐type MSAP markers, kinship‐log distance regressions were negative and statistically significant only for plant pairs in the same subpopulation, but did not reach significance for pairs in different subpopulations; and for *h*‐type MSAP markers, kinship‐log distance regressions were statistically nonsignificant in all cases (Table 3).

**Table 3:** Isolation‐by‐distance tests of spatial structuring of genetic (AFLP markers) and epigenetic (MSAP markers) diversity, performed by fitting separate kinship*‐*distance (log‐transformed) regressions for plant pairs with pair members in the same and different subpopulations. *P*‐values correspond to one‐sided tests of the hypothesis that the regression slope (*b*) was <0, and were obtained by permutations. Kinship‐distance regressions shown in Figure 5

Marker type	Pair members in same subpopulation: undisturbed		Pair members in same subpopulation: disturbed		Pair members in different subpopulations	
	*b* (SE)	*P*‐value	*b* (SE)	*P*‐value	*b* (SE)	*P*‐value
AFLP	−0.0084 (0.0031)	0.008	−0.0128 (0.0020)	<0.0001	−0.0071 (0.0029)	0.0004
MSAP *m*‐type	−0.0086 (0.0030)	0.009	−0.0041 (0.0019)	0.018	−0.0015 (0.0027)	0.22
MSAP *h*‐type	+0.0011 (0.0024)	0.66	+0.0033 (0.0030)	0.95	−0.0004 (0.0030)	0.41

**Figure 5:**
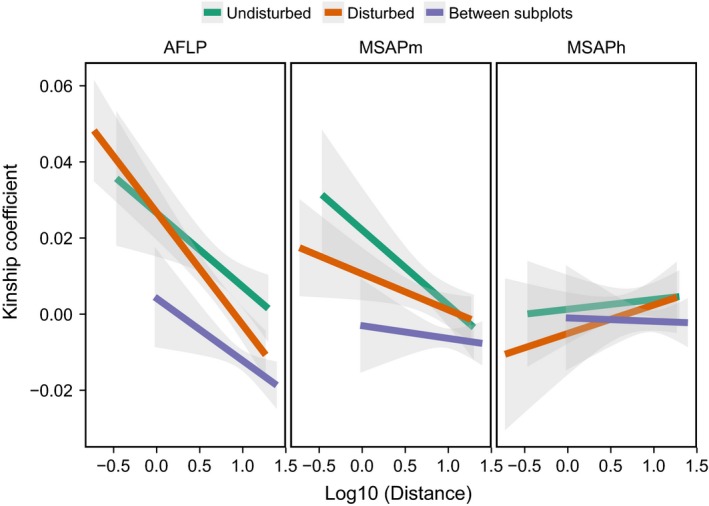
Least squares‐fitted regressions and 95% confidence intervals (shaded areas) for the relationships between kinship and log distance for pairs of *Lavandula latifolia* plants, broken down by marker type (three panels; MSAPm and MSAPh stands for *m*‐type and *h*‐type markers, respectively) and categories of plant pairs. Three classes of plant pairs were recognized (color‐coded lines in each panel), depending on whether plants in the pair were in the same or different subplots. With the definition of kinship used, negative kinship coefficients simply mean that the individuals involved are less related than random individuals drawn from the reference population (Hardy 2003).

Comparisons of fitted kinship‐log distance regressions clarify and extend these results (Fig. 5). Fine‐scale spatial structuring of genetic variation was essentially identical in the two subpopulations, as shown by the overlap of kinship‐log distance regression lines with their associated confidence intervals over the range of distances considered. Nevertheless, kinship of plant pairs from different subpopulations was lower than kinship of pairs in the same subpopulation. The pattern exhibited by regressions for *m*‐type MSAP markers was qualitatively similar to that of AFLP ones (Fig. 5), except for one important quantitative difference: the slope for the Disturbed subpopulation was shallower than the slope for the Undisturbed subpopulation, and the slope for the different‐subpopulations regression did not reach significance (Table 3). Flat regressions for *h*‐type MSAP markers confirmed the absence of isolation‐by‐distance structuring or differences between pair classes (Table 3).

## Discussion

### Correlates of disturbance

Ecological investigations on the effects of disturbance have concentrated on the impact of devastating agents such as fire or hurricanes, but milder perturbations may also have significant consequences for populations and communities (Lavorel et al. 1994; Herrera 1997; Eschtruth and Battles 2014). Manual removal of all *L. latifolia* plants from the Disturbed subplot was a comparatively mild disturbance, yet the plants that quickly reestablished there were ecologically, genetically, and epigenetically distinct from those that established by about the same time in the adjacent Undisturbed subplot. In addition, our results are consistent with the interpretation that genetic and epigenetic differences between plants in the two subplots subpopulations were largely the consequence of experimental disturbance. This is supported by the lower differentiation levels in the artificial test dataset and, particularly, by the results of Bayesian clustering. The abrupt linear transition between the two clusters obtained closely matched the demarcation line between subplots arbitrarily defined at the beginning of the experiment, 20 years before plants were sampled for this study. This finding renders extremely implausible the hypothesis that observed genetic and epigenetic differences between subpopulations were mainly the legacy of some pre‐existing within‐plot spatial gradient(s).

The reestablished subpopulation had distinctive demographic features relevant to the interpretation of this study. Prior to experimental setup in 1986, *L. latifolia* was the single dominant shrub across the whole study plot, and proportion of reproductive individuals and inflorescence production per plant were similar in the two arbitrarily defined subplots. Sudden disappearance of all plants from the experimental subplot produced an abrupt increase in irradiance at ground level and also, presumably, a reduction of competition for water and nutrients. These changes should have in the first place stimulated the germination of seeds present in the soil, and then enhanced the survival and early growth of seedlings, as reported for other *Lavandula* species from Mediterranean‐climate habitats (Herrera 1997; Sánchez and Peco 2007). Fast growth and early onset of reproduction were also two distinctive features of reestablished individuals in our disturbed subplot (Herrera and Jovani 2010), in agreement with previous studies on postdisturbance plant colonization (Cook and Lyons 1983; Scheiner and Teeri 1987; Dolan et al. 2008; Roberts et al. 2014). Rapid phenotypic changes following disturbance have been related to shifts in genetic composition of populations (Scheiner and Teeri 1987). Our results support this view, but also suggest that epigenetic shifts and release of epigenetic variation (e.g., via environmentally driven changes in cytosine methylation; Alonso et al. 2016) might also have contributed to these phenotypic changes.

Because of its faster ‘demographic pace’, the Disturbed subpopulation was able to catch up with the Undisturbed one (in terms of proportion of plants flowering and inflorescence production) only 10 years after disturbance. Over the next 10 years the two subpopulations became phenotypically undistinguishable even to the advised observer, and their reproductive parameters fluctuated annually in unison. By 2005, when leaf samples were collected for this study, plants from the Disturbed subpopulation were, on average, 5 years younger than those from the Undisturbed one. Age difference between plants from the two subpopulations might have contributed to their epigenetic divergence if cytosine methylation varied with age in adult *L. latifolia* plants. In 2014, those plants considered in this study still surviving in the Disturbed (*N *=* *12) and Undisturbed (*N *=* *19) subpopulations were resampled and MSAP analyses repeated. Results showed that epigenetic differences between subpopulations reported here persisted essentially unaltered almost one decade later, and that longitudinal changes exhibited by some plants were quantitatively negligible in relation to differences between subpopulations (C. M. Herrera and P. Bazaga, unpubl. data). Genetic and epigenetic differences between subpopulations found in this study should thus most likely be related to the contrasting ecological backgrounds prevailing during seedling emergence and early growth of plants rather than to slight differences in age.

### Genetic variation

Genetic diversity did not differ between subpopulations, but these did differ in multilocus genetic characteristics. The AMOVAs revealed significant differences, and up to six AFLP markers were nonrandomly distributed between subpopulations. Such genetic differences were partly due to an underlying isolation‐by‐distance pattern occurring across the whole plot (Meirmans 2012), as revealed by negative associations between kinship and distance (log‐transformed) for plant pairs within each of the two subpopulations and also for pairs in different subpopulations. The lower intercept of the kinship‐distance regression for pairs in different subpopulations, however, demonstrated that genetic differences between subpopulations were greater than would be expected from isolation by distance alone, since for a given distance kinship for plant pairs in different subpopulations was lower than for pairs in the same subpopulation. Results of Bayesian clustering clearly support this interpretation.

Isolation‐by‐distance tests provided cues on the possible mechanism accounting for observed genetic differences between subpopulations. The small‐scale isolation by distance of AFLP markers corroborates the expectation, based on earlier studies on seed dispersal and pollinator flight distances (Herrera 1987b), that gene dispersal is very restricted in *L. latifolia* (see also Sánchez and Peco 2007). Spatially restricted dispersal and germination of most seeds during the first few years in the soil (Herrera 2000), suggest that plants reestablished in the Disturbed subplot arose from seeds present in the soil at the time of disturbance and were the progeny of adult plants removed experimentally. Strong support for this idea is provided by the close coincidence between the kinship‐distance regressions for plant pairs within the Disturbed and Undisturbed subplots, showing that spatial patterning of genetic diversity in the Undisturbed subplot was soon replicated ex novo by plants reestablished in the Disturbed one. This finding suggests that, when disturbance was performed, seeds in the soil of the Disturbed subplot were genetically similar to their parent plants, as found in other species (Mahy et al. 1999; Honnay et al. 2008). Consequently, genetic differences between subpopulations 20 years after disturbance probably originated from divergent selection on non‐neutral AFLP markers (or linked to non‐neutral ones) induced by the contrasting environments experienced by plants in the two subpopulations during their early life stages. Shifts in selective regime following disturbance are probably widespread, as they have been often implicated in postdisturbance changes in herozygosity (Honnay et al. 2008; Roberts et al. 2014), but few studies have documented postdisturbance genetic shifts (Dolan et al. 2008; Banks et al. 2013).

### Epigenetic variation

Partial Mantel tests on dissimilarity matrices revealed that epigenetic differences between subpopulations were (statistically) unrelated to genetic differences. This finding, and the unrelatedness of AFLP and MSAP dissimilarity matrices, suggest that epigenetic variation was largely autonomous from genetic variation in the sample of *L. latifolia* plants studied, as found in other wild plants (Li et al. 2008; Paun et al. 2010; Herrera et al. 2016). Percent polymorphim of MSAP markers in the Disturbed subpopulation almost doubled that in the Undisturbed one, mainly because of the substantial increase in polymorphism of *h*‐type markers (CHG‐context methylation). In contrast, epigenetic differentiation between subpopulations was largely caused by *m*‐type markers (CG‐context methylation). Since spatial genetic patterns discussed above deny a major role of immigrant seeds in the reestablishment of the Disturbed subpopulation, the latter's epigenetic differentiation and increased diversity most likely resulted from a combination of isolation‐by‐distance effects, postdisturbance divergent selection and appearance of new epigenetic variants through transgenerational modification of methylation marks. New epigenetic variants could have arisen spontaneously (e.g., via unfaithful meiotic transmission, leading to parent‐offspring differences; Herrera et al. 2013; Lauria et al. 2014) and/or in response to the environmental changes brought about by disturbance (e.g., somatic modifications between the seed and adult plant stages; Boyko and Kovalchuk 2008; Feng et al. 2010).

In the Undisturbed subpopulation, the kinship‐distance regression for *m*‐type markers was virtually identical to that for AFLP markers. Since dispersal patterns of nuclear genetic and epigenetic markers should by definition be identical, a parsimonious interpretation of this result is that in the Undisturbed subpopulation the transgenerational inheritance of *m*‐type and AFLP markers had been sufficiently similar during a number of prior generations as to produce identical isolation‐by‐distance signatures (Herrera et al. 2016). In this scenario, the slight increase in polymorphism of *m*‐type markers in the Disturbed subpopulation should be due to modest modifications of methylation marks induced by disturbance at the somatic stage (i.e., from seed to seedling or adult plant), while the significant differentiation between subpopulations would reflect divergent selection on non‐neutral *m*‐type markers (or linked to non‐neutral ones). The shallower slope of the kinship‐distance regression for *m*‐type markers in the Disturbed subpopulation is compatible with these interpretations, since both selection and reduced heritability act reducing the strength of isolation‐by‐distance patterns (Epperson 1990, 2005; Ley and Hardy 2013; Herrera et al. 2016).

Variation of *h*‐type markers was not spatially structured at any subpopulation, as shown by flat kinship‐distance regressions. Given the highly significant isolation‐by‐distance patterns shown by both AFLP and *m*‐type markers in the Undisturbed subpopulation, this contrasting result can only be interpreted as reflecting inherently low transgenerational inheritance of *h*‐type markers, which would have precluded the appearance of spatial structuring even in the face of restricted dispersal (Ley and Hardy 2013; Herrera et al. 2016). Increased *h*‐type marker polymorphism in the Disturbed subpopulation most likely arose from a burst of somatic unstability in this type of methylation marks induced by the sudden change in ecological factors, such as water and light availability, which are known to influence pattern and extent of cytosine methylation in plants (Alonso et al. 2016). Statistical nonsignificance of AMOVAs, along with the underrepresentation of *h*‐type markers among those nonrandomly distributed between subpopulations, point to absence of selection and predominantly random nature of the new *h*‐type methylation variants induced by disturbance.

### Implications and future directions

Mild ephemeral disturbances can have long‐lasting impacts on plant communities (e.g., promoting the invasion of exotic species; Eschtruth and Battles 2014). Our results likewise suggest that, despite mild intensity and quick recovery, experimental disturbance left lasting genetic and epigenetic signatures on the next generation of adult *L. latifolia* plants. Studies on the spatial structure of genetic and epigenetic variation in long‐lived plants might therefore be misled by ‘invisible scars’ left by past disturbances on seemingly homogeneous populations. Spatial boundaries of old unrecognized disturbances, similar to the abrupt linear transition between subplots revealed here by Bayesian clustering (Fig. 4), might be taken erroneously for barriers to gene flow.

Results of this study also highlight other aspects that should be considered by future investigations on the effects of ecological disturbance and, more generally, by investigations in the currently expanding field of ecological epigenetics (Kilvitis et al. 2014). Prominent among these is the need of long‐term monitoring of postdisturbance changes (Rice and Jain 1985; Eschtruth and Battles 2014). In communities dominated by long‐lived woody plants regenerating from seeds, studies spanning at least one generation are essential to understand the possible mechanisms linking disturbance with functional, genetic and epigenetic diversity. In *L. latifolia*, genetic and epigenetic differences between subpopulations ran parallel to demographic differences (faster growth, earlier reproduction in the reestablishing population). This suggests that disturbance enhanced intraspecific functional diversity through increased genetic and epigenetic diversity at the population level. AMOVAs revealed that, 20 years after disturbance, differences between subpopulations contributed 2.5% and 1.4% to local genetic and epigenetic diversity (Disturbed + Undisturbed subpopulations combined), respectively, which is remarkable given the restricted spatial scale considered.

The contrasting results obtained for different MSAP marker types is another aspect deserving consideration by future studies on natural epigenetic variation of nonmodel plants. Differential spatial patterns for different marker types were also reported by Schulz et al. (2014) in an investigation on geographical and habitat‐dependent structuring of epigenetic variation in the perennial herb *Viola elatior*. It is tempting to speculate that these differences are related to differences between cytosine methylation in CHG (*h*‐type markers) and CG (*m*‐type markers) contexts in maintenance mechanisms and predominant genomic location (genes vs. transposable elements) (Cokus et al. 2008; Lister et al. 2008; Vanyushin and Ashapkin 2011; Osabe et al. 2014). Irrespective of mechanisms, our results emphasize the importance of adopting scoring approaches in future MSAP‐based ecological epigenetics studies that allow to differentiate spatial structure, variability levels, transgenerational constancy and phenotypic correlates of cytosine methylation marks in different genomic contexts.

An increasing number of studies show that epigenetic variation in natural plant populations may be spatially structured at the landscape and regional levels, but explicitly spatial methods have been only rarely used to examine fine‐scale spatial structuring (Herrera et al. 2016). Application of explicitly spatial methods to MSAP marker data has proven useful here on different grounds. For one thing, tests of expectations under isolation‐by‐distance models assisted in the identification of the possible mechanisms generating epigenetic differences between subpopulations. We also found, apparently for the first time, that fine‐scale spatial structuring of one class of MSAP markers conformed to the same isolation‐by‐distance model originally developed for genetic variation, and that their isolation‐by‐distance parameters were nearly indistinguishable from those for genetic variation. Comparisons of fine‐scale spatial patterning of natural genetic and epigenetic variation may become an useful tool for gathering information on the challenging issues of long‐term transgenerational stability of epigenetic marks and epigenetic adaptive responses to environmental variation under natural field conditions (Herrera et al. 2016).

## Data Accessibility

AFLP and MSAP data, and spatial coordinates of sampled plants, deposited at DRYAD doi:10.5061/dryad.v4192.

## Conflict of Interest

None declared.

## Supporting information


**Table S1.** Primer combinations, scoring errors, number of markers, and polymorphism levels for AFLP and MSAP analyses.

**Table S2.** AFLP and MSAP markers nonrandomly distributed between Undisturbed and Disturbed subpopulations.
