# Supplementary material for: Genetic and epigenetic divergence between disturbed and undisturbed subpopulations of a Mediterranean shrub: a 20‐year field experiment
Source: Ecol Evol. 2016 May 9;6(11):3832–47. doi: 10.1002/ece3.2161 (PMC5513313; doi:10.1002/ece3.2161)
Supplement: Supplementary file 1 — Table S1. Primer combinations, scoring errors, number of markers, and polymorphism levels for AFLP and MSAP analyses. [file ECE3-6-3832-s001.doc]

**Table S1.** Primer combinations, number of fragments obtained in the size range 150-500 base pairs, polymorphism level, and estimates of scoring error rates, in the amplified fragment length polymorphism (AFLP) and methylation-sensitive amplified polymorphism (MSAP) analyses of the *Lavandula latifolia* plants considered in this study. In MSAP analyses, fragments were transformed to two marker classes following the ‘Mixed Scoring 2’ scheme of Schulz et al. (2013), corresponding to ^HMe^CCG methylation (*h*-type markers) and ^HMe^CG + ^Me^CG methylation (*m*-type markers).

S1.1. AFLP analyses

| Primer combination | Scoring error rate (%) ¶ | Fragments | Polymorphism (%) * |
| --- | --- | --- | --- |
| *Eco* AAT– *Mse* CGT | 2.06 | 18 | 50.0 |
| *Eco* ACA– *Mse* CAT | 1.57 | 40 | 40.0 |
| *Eco* ACC– *Mse* CGT | 1.95 | 38 | 39.5 |
| *Eco* AGG– *Mse* CCT | 3.34 | 35 | 37.1 |
| *Pst* AA– *Mse* CGT | 2.61 | 23 | 47.8 |
| *Pst* AC– *Mse* CAC | 2.86 | 22 | 27.3 |
| *Pst* AC– *Mse* CCT | 1.12 | 33 | 39.4 |
| *Pst* AG– *Mse* CGT | 2.18 | 21 | 76.2 |
| All combined | 2.16 | 230 | 43.0 |

¶ Calculated from the 35 plants (23.5% of total) that were assayed twice as 100 x (number of discordant scores on two analyses) / (number of scored markers x number of individuals).

* A marker was considered polymorphic if >2% of samples showed a variant score.

S1.2. MSAP analyses

|  |  |  |  | *h*-type markers |  |  | *m*-type markers |  |
| --- | --- | --- | --- | --- | --- | --- | --- | --- |
| Primer combination | Scoring error rate (%) ¶ | Fragments § |  | Total | Polymorphism (%) * |  | Total | Polymorphism (%) * |
| *Hpa*-*Msp* TA– *Mse* CTA | 3.49 | 60 (91.7) |  | 52 | 53.8 |  | 39 | 69.2 |
| *Hpa*-*Msp* TC– *Mse* CGC | 2.44 | 41 (95.1) |  | 38 | 52.6 |  | 25 | 76.0 |
| *Hpa*-*Msp* TG– *Mse* CTA | 4.02 | 35 (80.0) |  | 32 | 40.6 |  | 21 | 71.4 |
| *Hpa*-*Msp* TT– *Mse* CAC | 2.28 | 37 (67.6) |  | 31 | 48.4 |  | 37 | 27.0 |
| All combined | 3.09 | 173 (85.0) |  | 153 | 49.7 |  | 122 | 58.2 |

* A marker was considered polymorphic if >2% of samples showed a variant score.

§ In parentheses, percentage of fragments leading to *h*-type or *m*-type markers.

¶ Calculated from the 32 plants (21.5% of total) that were assayed twice, and computed as the ratio of the number of discordant scores in the two analyses (all plants and markers, and the two enzyme pairs, combined) to twice the product of the number of plants by the number of scored markers.

**References**

Schulz B, Eckstein RL, Durka W. 2013. Scoring and analysis of methylation-sensitive amplification polymorphisms for epigenetic population studies. Molecular Ecology Resources 13:642–653.
