# Supplementary material for: Genetic and epigenetic divergence between disturbed and undisturbed subpopulations of a Mediterranean shrub: a 20‐year field experiment
Source: Ecol Evol. 2016 May 9;6(11):3832–47. doi: 10.1002/ece3.2161 (PMC5513313; doi:10.1002/ece3.2161)
Supplement: Supplementary file 2 — Table S2. AFLP and MSAP markers nonrandomly distributed between Undisturbed and Disturbed subpopulations. [file ECE3-6-3832-s002.doc]

**Table S2.** Genetic (AFLP) and epigenetic (MSAP) markers that either were significantly associated with (marker x subpopulation association analyses), or had significant predictive value to discriminate between (Random Forests analysis), the two *Lavandula latifolia* subpopulations sampled in 2005 from the Undisturbed and Disturbed subplots. Markers are identified by primer combination and size (base pairs). For MSAP markers, the *m* and *h* prefixes denote ^HMe^CG + ^Me^CG methylated and ^HMe^CCG methylated marker types, respectively, according to the ‘Mixed Scoring 2’ scheme of Schulz et al. (2013). Since the main advantage of Random Forests classification over marker-by-marker separate association analyses resides in its ability to identify markers whose classificatory value may be effected through indirect interactions with others, the combined data matrix of polymorphic AFLP and MSAP markers was used as the input for this analysis.

|  | Analytical approach |  |
| --- | --- | --- |
| Marker type | Marker x subpopulation association (two-way contingency tables) * | Random Forests classification (Boruta algorithm) ¶ |
| AFLP: | *Eco*ACC–*Mse*CGT_314 (7.7e-06) *Eco*AGG–*Mse*CCT_493 (1.2e-04) *Eco*ACA–*Mse*CAT_247 (2.1e-04) *Eco*ACA–*Mse*CAT_305 (4.4e-03) *Eco*ACC–*Mse*CGT_346 (6.8e-03) *Eco*ACC–*Mse*CGT_215 (9.8e-03) | *Eco*ACC–*Mse*CGT_314 (100) *Eco*AGG–*Mse*CCT_493 (100) *Eco*ACA–*Mse*CAT_247 (100) |
| MSAP: | *mHpa*/*Mse*TA–*Msp*CTA_364 (8.5e-05) *mHpa*/*Mse*TC–*Msp*CGC_355 (1.2e-04) *mHpa*/*Mse*TC–*Msp*CGC_168 (5.9e-04) *mHpa*/*Mse*TC–*Msp*CGC_269 (2.7e-03) *mHpa*/*Mse*TG–*Msp*CTA_268 (3.6e-03) | *mHpa*/*Mse*TA–*Msp*CTA_364 (100) *mHpa*/*Mse*TC–*Msp*CGC_355 (100) *hHpa*/*Mse*TA–*Msp*CTA_316 (99.3) *mHpa*/*Mse*TC–*Msp*CGC_168 (97.3) |

* *P*-values for the significant marker x subpopulation associations are shown in parentheses, which were obtained by applying separate Fisher-exact probability tests for each marker (presence-absence) x subpopulation (Undisturbed-Disturbed) two-way contingency table.

¶ In parentheses the proportion (%) of *N* = 300 independent runs of the Boruta algorithm in which a given marker was ‘confirmed’ as having significant predictive (classificatory) value to discriminate between the two *L. latifolia* subpopulations studied. Only markers which were ‘confirmed’ in ≥ 95% of independent Boruta runs, whose predictive value was therefore most robust to random variations inherent to the classification algorithm, are listed.

**References**

Schulz, B., Eckstein, R.L., Durka, W. 2013. Scoring and analysis of methylation-sensitive amplification polymorphisms for epigenetic population studies. Molecular Ecology Resources 13:642–653.
